# Methods for cell isolation and analysis of the highly regenerative tunicate *Polycarpa mytiligera*

**DOI:** 10.3389/fcell.2023.1274826

**Published:** 2023-10-11

**Authors:** Tal Gordon, Noam Hendin, Omri Wurtzel

**Affiliations:** ^1^ The School of Neurobiology, Biochemistry and Biophysics, The George S. Wise Faculty of Life Sciences, Tel Aviv University, Tel Aviv, Israel; ^2^ Sagol School of Neuroscience, Tel Aviv University, Tel Aviv, Israel

**Keywords:** cell isolation, autofluorescence, flow cytometry, regeneration, marine model organisms

## Abstract

**Background:** *Polycarpa mytiligera* is the only molecularly characterized solitary ascidian capable of regenerating all organs and tissue types. The cellular basis for regeneration in *P. mytiligera* is largely unknown, and methods for isolating live cells from this species for functional analyses are unavailable.

**Results:** Here, we developed a method for isolating live cells from *P. mytiligera*, overcoming major experimental challenges, including the dissociation of its thick body wall and native cellular autofluorescence. We demonstrated the applicability of our approach for tissue dissociation and cell analysis using three flow cytometry platforms, and by using broadly used non-species-specific cell labeling reagents. In addition to live cell isolation, proof-of-concept experiments showed that this approach was compatible with gene expression analysis of RNA extracted from the isolated cells, and with *ex vivo* analysis of phagocytosis.

**Conclusion:** We presented efficient methods for cell purification from a highly regenerative ascidian, which could be transferable to diversity of non-model marine organisms. The ability to purify live cells will promote future studies of cell function in *P. mytiligera* regeneration.

## Background

Ascidians (Phylum: Chordata, Class: Ascidiacea) are marine filter feeding chordates, and are part of the sister group of vertebrates (Delsuc et al., 2006). Ascidians are broadly used in ecological (Shenkar and Swalla, 2011; Rocha et al., 2012; Ruli and Tapilatu, 2020), cellular (Ryan et al., 2016; Rosental et al., 2018), and molecular research (Harafuji et al., 2002). Colonial ascidians reproduce both sexually and asexually and can regenerate any missing body part (Kowarsky et al., 2021; Blanchoud et al., 2018). By contrast, solitary ascidians only reproduce sexually and have a varying regeneration capacity (Gordon et al., 2019; Kassmer et al., 2019). *Polycarpa mytiligera* is the only known solitary ascidian species that can regenerate any body tissue, making it an attractive candidate for functional studies of regeneration (Gordon et al., 2021). Recent studies have characterized regeneration in *P. mytiligera* and have established key techniques for molecular analysis: 1) production of a transcriptome assembly (Gordon et al., 2022; Hendin et al., 2022); 2) characterization of gene expression during regeneration (Gordon et al., 2022) and injury response (Hendin et al., 2022); 3) optimization of methods for analyzing gene expression by fluorescent *in situ* hybridization (FISH) or immunofluorescence in tissue sections and in whole mounts (Gordon et al., 2022; Hendin et al., 2022); and 4) DNA metabolic labeling for assessing S-phase progression (Gordon et al., 2021). Application of these methods has revealed cellular responses to injury and regeneration. For example, *P. mytiligera* regeneration is characterized by increased cell proliferation as observed by metabolic DNA labeling (Gordon et al., 2021), and cells expressing *BMP1* are detectable in the injured tunic 12 h following injury (Hendin et al., 2022).

Despite progress in optimization of molecular methods, techniques for isolation of live cells are missing in *P. mytiligera*. Therefore, isolation of cellular populations that are implicated in regeneration, such as stem cells, phagocytes or other immune cells (Lauzon et al., 2013; Londono et al., 2020; Mamilos et al., 2023), and analysis of their function are limited. Extraction of live cells involves both isolation of cells from tissues, and cell purification using Fluorescence-Activated Cell Sorting (FACS). Cell isolation requires tissue-specific optimization, based on the tissue characteristics, including tissue composition, availability, and state (Petit et al., 2013; Donaghy et al., 2017; Rosental et al., 2017; Qarri et al., 2022). Optimization of both the mechanical dissociation of the tissue and subsequent enzymatic digestion, if applied, is critical for obtaining true representation of the cellular populations that are found in the tissue (Petit et al., 2013; Rosental et al., 2017). Severe mechanical dissociation or harsh enzymatic treatment are often detrimental to the sample integrity and viability of sensitive cell types. By contrast, inefficient tissue disruption could result in poor cellular extraction from tissues (Petit et al., 2013). Therefore, it is necessary to empirically determine the optimal conditions for tissue dissociation prior to further processing.

Tissue dissociation generates a mix of cell populations, as well as cellular and non-cellular debris. Purification of live cells requires distinguishing between these components. This is often achieved by using FACS to separate cells based on their morphological properties (e.g., particle size and granularity) and by using functional and genetic fluorescent markers. Cells from marine organisms often have naturally occurring fluorescence (i.e., autofluorescence) (Swalla et al., 1994; Brown et al., 2009; Serrato et al., 2022). Therefore, application of fluorescent reagents for FACS requires prior assessment of the inherent fluorescent properties of the sample. Moreover, biological properties of the sample also influence the choice of fluorescent reagents compatible with cell purification (Rosental et al., 2018).

Cell extraction from *P. mytiligera* is challenging. Adult animals have opaque tissues and are covered by a thick, tough, integumentary tissue known as tunic. The tunic is composed of cellulose, collagens and other extracellular-matrix proteins, vasculature, and free cells (Smith and Dehnel, 1971; Hirose, 2009). The body wall epidermis is tough and highly pigmented, and internal tissues are fibrotic. Moreover, the tunic is covered by epibionts, such as algae and invertebrates (Shenkar and Gordon, 2015). Therefore, efficient cell purification strategies are required for overcoming these challenges.

Here, we developed a method for dissociating *P. mytiligera* tissues and extracting cells for further analysis. We analyzed the autofluorescence properties of the dissociated tissues using different flow cytometry approaches, and optimized a strategy for isolating live cell populations based on non-species-specific fluorescent markers. We demonstrated the utility of fluorescent labeling reagents on three flow cytometry platforms, including analyzer, sorter, and imaging flow cytometer. Finally, we demonstrated several potential uses of this method, by extracting RNA from FACS-purified cells and profiling their gene expression, and by performing proof-of-concept *ex vivo* phagocytosis assay. Our work facilitates cell purification from *P. mytiligera*, which is necessary for development of functional cell assays, and therefore advances the analysis of the cellular basis of *P. mytiligera* regeneration.

## Results

### Cell extraction from *P. mytiligera* tissues

**FIGURE 1:**
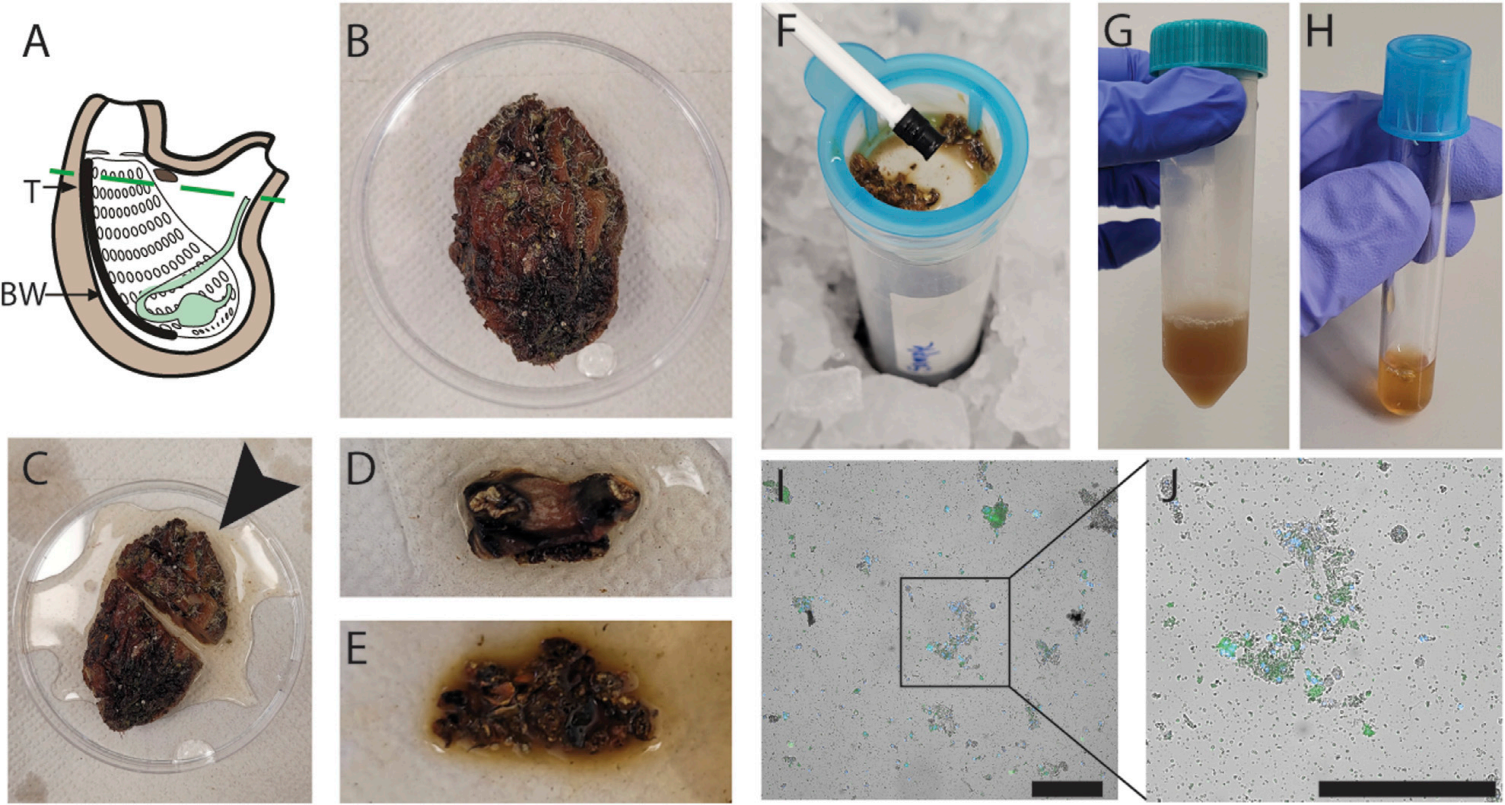
Optimization of cell extraction from *P. mytiligera*. **(A)** Diagram of adult *P. mytiligera* showing the body region isolated for cell extraction (region above the dashed line). T, tunic; BW, body wall. **(B)** Photo of an adult *P. mytiligera* before dissection showing its opaque tunic and covering epibionts. **(C)** Separation of anterior region (black arrow) for cell isolation, prior to removal of covering tunic. **(D)** Isolated anterior region following tunic removal. **(E)** Tissue fragments following fine dicing using a razor blade. **(F)** Mechanical filtration of finely diced fragments with a 40 µm filter using a plunger. **(G, H)** Resultant cell suspension prior to **(G)** and following **(H)** repeated centrifugation for cell collection. **(I, J)** Live cells obtained using optimal cellular extraction parameters. Cells are labeled with nuclear (Hoechst) and viability (calcein) labels, blue and green, respectively. Debris and cell aggregates are detectable as well. Square **(I)** indicates higher magnification of a region containing cells shown in panel **(J)**. Scale = 50 µm.

To isolate cells from adult *P. mytiligera*, we surgically separated the covering tunic from the underlying tissues. We isolated tissues from the anterior body region, including the neural complex, oral and atrial siphons, branchial basket, and the surrounding body wall (Figures 1A–C). Isolated tissues (<3 cm long) were diced to small fragments (∼1 mm) using a razor on ice (Figures 1D,E). Then, the fine fragments were washed in medium (Methods). The fragments were then subjected to enzymatic treatment and filtered using a mesh. Alternatively, fragments were filtered using a mesh without enzymatic treatment. Cells were collected by centrifugation, and additional filtration steps, resulting in a pigmented cell suspension (Figures 1F–H; Methods). Finally, cells were labeled with a viability dye (propidium iodide, PI, or calcein; Supplementary Figure S1), and cell viability was assessed by microscopy. PI^+^ cells were considered dead or dying cells, and calcein^+^ cells were considered alive, based on their activity on calcein (Methods). We tested different medium conditions and enzymatic treatments for optimizing the cell extraction and cell viability (Methods). The media tested included artificial seawater (ASW) recipes, which were previously used for marine invertebrate cell isolation (Levy et al., 2021; Wang et al., 2018) or PBS, in a range of pH and media osmolarities. Cell viability in most media conditions was very poor, with >90% non-viable cells, as indicated by positive PI labeling (Supplementary Figure S1; Methods). Mild enzymatic treatment with collagenase I and no-enzymatic treatment resulted in the best viable cell recovery (Figures 1I,J; Supplementary Figure S1; Methods). However, omitting enzymatic treatment has reduced the length of the procedure, and the handling of the samples, and it was therefore favored for subsequent experiments.

### Analysis of autofluorescence of *P. mytiligera* cells

**FIGURE 2:**
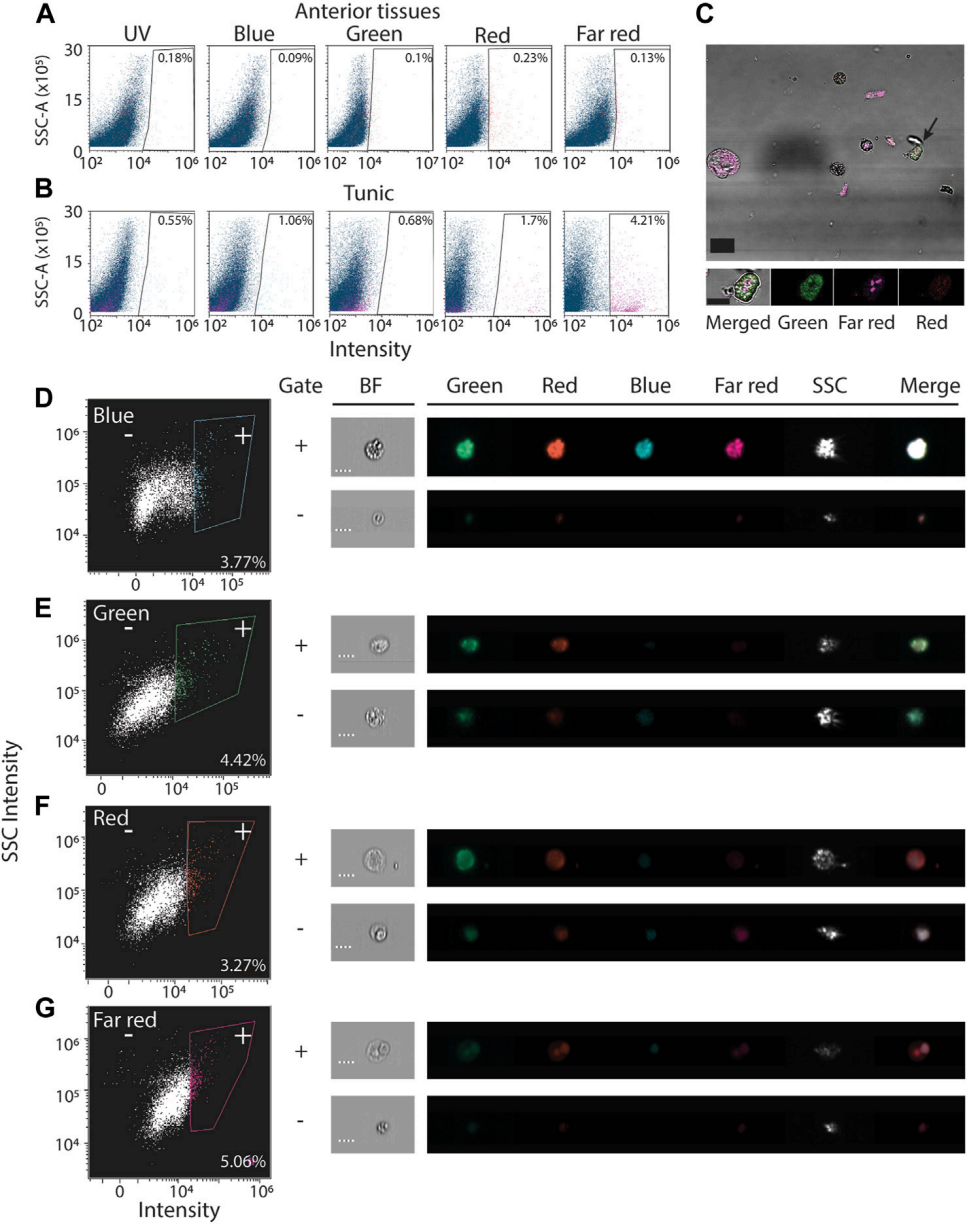
Autofluorescence in isolated *P. mytiligera* cells. **(A, B)** Shown are representative flow cytometry plots of cells isolated from anterior tissues **(A)** or tunic **(B)** selected from three independent replicates of the experiment. *X*-axis shows intensity measured using laser and filter corresponding to the label and *Y*-axis is the measured side scatter. Gates defining cells having autofluorescence are shown with the fraction of cells detected in the gate out of all cells. The label is the average of three experiments. **(C)** Shown are cells having autofluorescence imaged using fluorescence microscopy (Methods). Tissue was dissociated and imaged without fluorescent labeling. Black arrow (top panel) indicates the cell in the high magnification image (bottom). scale = 20 and 10 μm, top and bottom panels, respectively. **(D–G)** Autofluorescence analysis using an imaging flow cytometer (Methods). Left panels show representative flow cytometry analyses. Right panels: shown are images of cells registered during the ImageStream analysis. Shown is the fluorescence intensity in every channel and a bright field image (BF). Bounding rectangles were added to the captured images for clarity. Plus and minus symbols represent inclusion in or exclusion from the gate, respectively. Label indicates the fraction of cells included in the positive (+) gate. Scale = 7 μm; SSC-A, side scatter area; SSC, side scatter.

Isolated cells from ascidians frequently display autofluorescence (Swalla et al., 1994; Brown et al., 2009; Serrato et al., 2022). To assess the extent of autofluorescence in *P. mytiligera*, we analyzed unlabeled cells extracted from tissues using flow cytometry and microscopy (Methods). Flow cytometry indicated that a small subset (<1%) of the unlabeled cells extracted from the upper body region, without the tunic, has detectable autofluorescence in the tested wavelengths (Figure 2A). This indicated that many fluorophores were likely compatible with *P. mytiligera* cells for cell purification. Similarly, we assessed autofluorescence in extracts from the tunic of *P. mytiligera* (Figure 2B). We found higher levels of autofluorescence in every tested wavelength, and particularly in the far-red range (excitation/emission, 638/660 nm), where 4.21% of the cells displayed autofluorescence (average of three replicates; Figure 2B).

The detected autofluorescence could originate from a distinct cell population. We used two strategies to validate the presence of cells having autofluorescence. First, we imaged unlabeled dissociated cells from the body wall, and detected autofluorescence in both the green and far-red channels (Figure 2C; Methods). These cells appeared granular, in agreement with previous reports of autofluorescence from granular ascidian cells (Brown et al., 2009; Serrato et al., 2022). Second, we used a platform combining flow cytometry and imaging (ImageStream; Methods) for detecting autofluorescence in cells in unlabeled body wall samples (Figure 2D). In agreement with traditional flow cytometry and microscopy (Figures 2A,C), we detected autofluorescence in cells, although in this analysis their abundance was higher (Figures 2D–G). This higher prevalence could reflect differences in sensitivity of the instrument, or availability of filters and excitation lasers for each instrument. In this analysis, registered events were imaged during detection by the flow cytometer (Figures 2D–G).

### Optimization of purification of live cells using FACS

**FIGURE 3:**
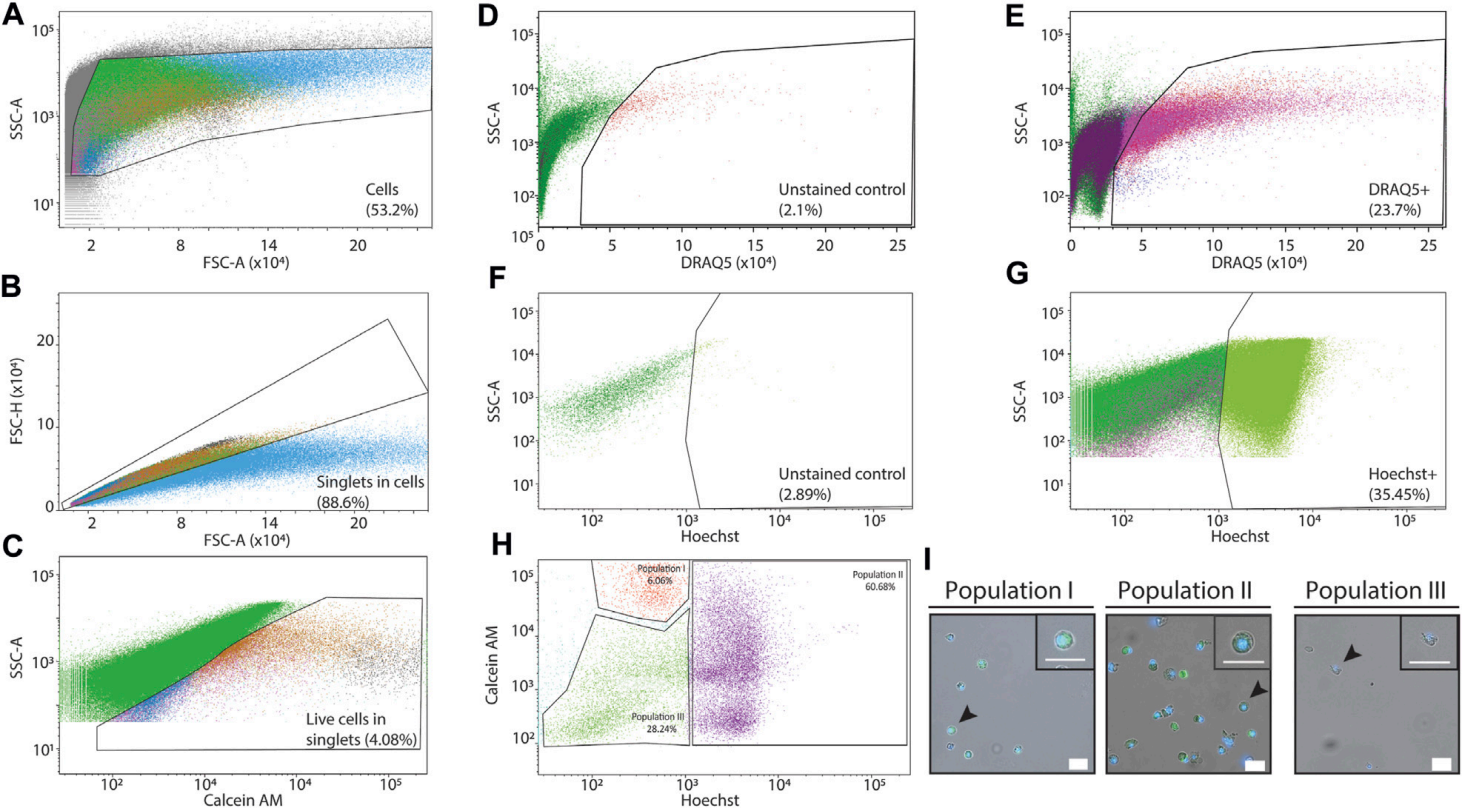
Purification of *P. mytiligera* cells by FACS. **(A–H)** Shown are flow cytometry analyses of labeled and unlabeled samples. *X*-axis and *Y*-axis show signal intensity (arbitrary units, A.U.) as measured by FACS in the channel shown in the label (Methods). Detection filters for different channels and wavelengths were as follows: Hoechst: 405 nm, 450/40 band pass (BP), calcein green (AM): 488 nm, 502 long pass (LP) 530/30 BP, and DRAQ5: 640 nm, 690 LP 730/45 BP. **(A)** Gating strategy for eliminating non-cellular particles using side scatter area (SSC-A) and forward scatter area (FSC-A). **(B)** Separating aggregates and large particles from single cells (singlets) using FSC-A and FSC height (FSC-H). **(C)** Using calcein labeling and SSC-A for isolating live cells from the singlet channel. **(D, E)** Analysis of signal in the DRAQ5 emission wavelength in unlabeled **(D)** and DRAQ5-labeled **(E)** samples has shown over 10-fold enrichment of live single cells (singlets) in the labeled sample. **(F, G)** Analysis of signal in the Hoechst emission wavelength in unlabeled **(F)** and Hoechst-labeled **(G)** samples has shown over 12-fold enrichment of live single cells (singlets) in the labeled samples. **(H, I)** Detection of putative cell populations by combining Hoechst and calcein labeling. The cells from the different gates **(H)** were sorted by FACS. Then, the purified samples were examined using confocal microscopy (I; Methods). Samples having high Hoechst and/or calcein (population I and II) showed an abundance of cells with little debris. By contrast, cells having low Hoechst and calcein in the flow cytometry analysis (population III) were sparse. Scale = 20 µm.

Purification of live cells from a dissociated tissue requires elimination of debris and dead cells. We dissociated *P. mytiligera* tissues to cell suspension (Methods). Prior to cell purification we found an abundance of cellular debris and cell aggregates (Figures 1I,J). We separated debris and removed cell aggregates by using side scatter area (SSC-A), and forward scatter area and height, FSC-A and FSC-H, respectively (Figures 3A,B). We used labeling reagents to separate live and dead cells. First, we used calcein to detect live cells (Figure 3C). Then, we labeled nuclei using DRAQ5 and Hoechst for detecting nucleated particles (Figures 3D–G). Comparison of unlabeled (Figures 3D,F) and labeled samples (Figures 3E,G) showed strong enrichment (>10-fold) for nucleated particles following labeling. Combining Hoechst and calcein was particularly useful for isolating live cells (Figure 3H), and distinguished between at least three potential cell populations. We isolated the potential three cell populations using FACS and imaged the cells using confocal microscopy (Methods; Figures 3H–I). We found that the two populations showing high intensity of calcein emission were indeed cells, based on assessment of nuclear and cytoplasmic morphologies and size (Figure 3I). By contrast, cells having low Hoechst and low calcein, were very sparse on the slide. The cell boundary of this sparse population was ruffled, the cells contained blobs, and the low Hoechst signal was detectable throughout the cell. These observations indicated that the third population might have suffered cell loss prior to imaging by microscopy (Figure 3I).

### Application of imaging flow cytometry for detection of cell types

**FIGURE 4:**
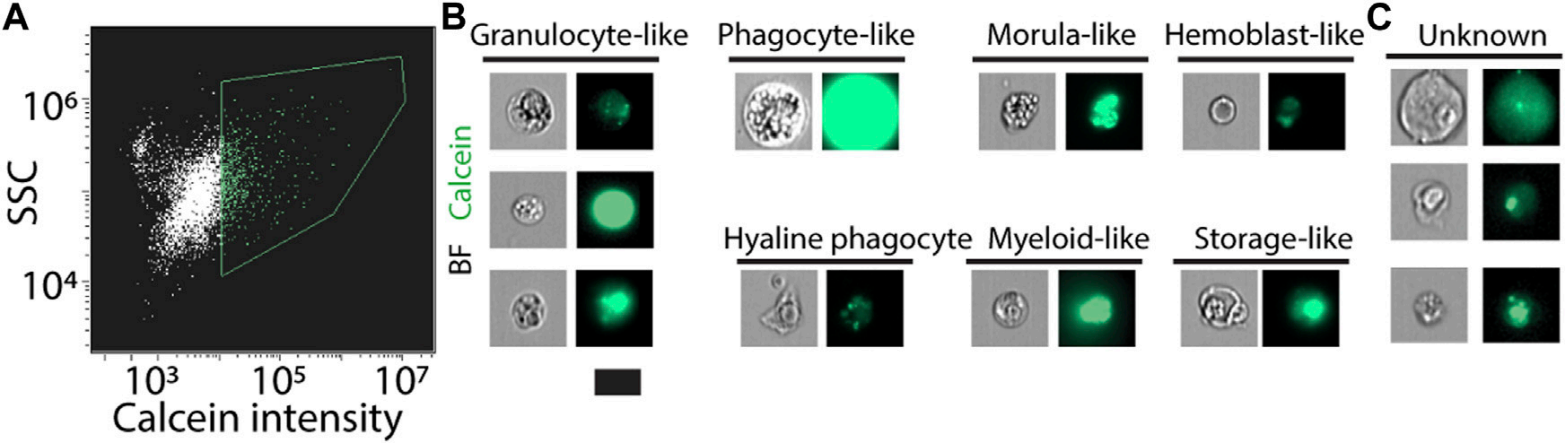
Live cell detection using an imaging flow cytometer. **(A)** Live cells were detected using calcein labeling and assessment of SSC (Methods). Green contour shows gate used for selecting the calcein positive cells. **(B, C)** Images of the analyzed cells were acquired by the instrument. **(B)** Shown are cells that have morphology that resembled known ascidian cell types. Enrichment for hematopoietic cell types was observed, potentially because of preferential isolation of these cells. **(C)** Shown are cells that we did not classify as resembling a cell type. Scale = 10 µm.

We applied the labeling strategies described above and used an imaging flow cytometer (Figure 4A; Methods). The instrument captures bright field and fluorescence images for the analyzed flow cytometry events. We manually analyzed the images and extracted photos of cells with distinct morphologies, which resembled known ascidian cell types (Figure 4B) (Arizza and Parrinello, 2009; Cima et al., 2016; Cima et al., 2017; Zeng et al., 2022). We observed enrichment in cell morphologies that are typical to hematopoietic cell types, suggesting an enrichment for such cell types in this flow cytometry approach. This could be the outcome of a less complex morphology or better compatibility of such cells with the tissue dissociation protocol. We also found cells having morphologies, which we could not associate with known cell types (Figure 4C). This analysis indicated that our approach indeed recovered multiple cell types with an intact cell morphology.

### RNA analysis from sorted *P. mytiligera* cells

**FIGURE 5:**
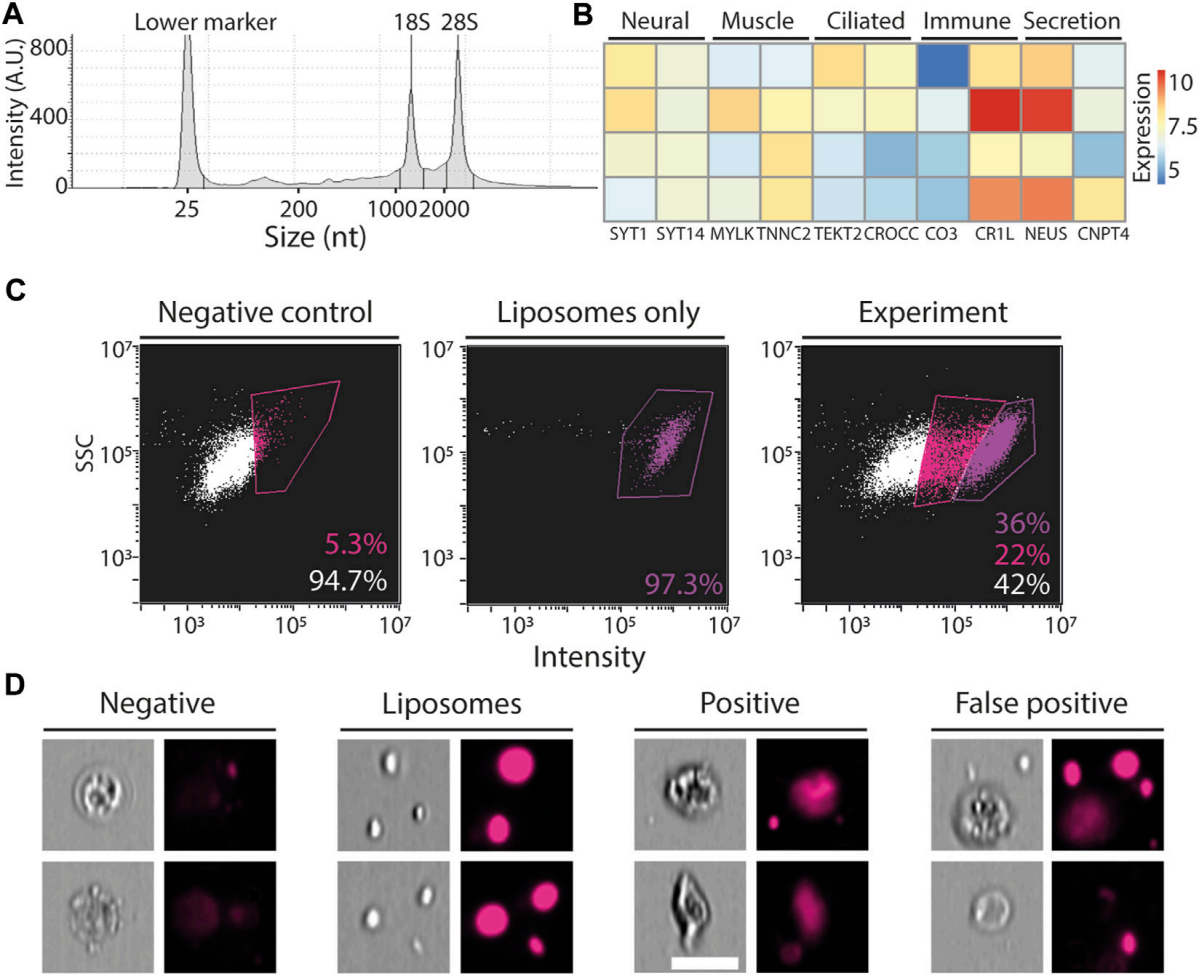
Application of FACS. **(A)** Shown is a representative microcapillary electrophoresis analysis of RNA that was extracted from ∼10,000 FACS-purified cells (Methods). A histogram of RNA species abundance showed that the 18S and 28S ribosomal RNAs remained intact, indicating that the integrity of the isolated RNA from the purified cells was high. **(B)** Expression of genes that have been associated with expression in different tissues, in other model systems, is shown. Blue and red, low and high gene expression, respectively. Expression was calculated using the variance-stabilizing transformation in DESeq2 (Love et al., 2014). Rows represent independent RNAseq libraries that were prepared from purified cells, columns represent genes, and are annotated with UNIPROT gene symbols. **(C)** Flow cytometry plots showing fluorescence in far red (642–745 nm) and SSC, X and Y-axes, respectively, in three samples: left, negative, cells that were not incubated with fluorescent liposomes; middle, liposomes without cells; right, experiment, cells incubated with fluorescent liposomes. The negative (unlabeled) and liposome only samples were used for determining the thresholds for background and positive signal. **(D)** Representative images from the imaging flow cytometer showing events captured in this analysis. Negative: cells showing low fluorescence in the far-red channel following incubation with fluorescent liposomes. Liposomes: aggregates of liposomes detected by the imaging flow cytometer. Positive: cells incubated with fluorescent liposomes showing high intensity signal localized in the cell body. False positive: cells detected as positive for high intensity signal, and that by inspection of the photo were found to have the liposomes attached externally to the cell body. Scale = 10 µm.

We next tested whether the FACS-purified cells were compatible with standard RNA extraction. *P. mytiligera* tissues were dissociated. Then, cell suspensions of total live cells were subjected to FACS purification directly into the extraction buffer, TRIzol LS (Methods). RNA extraction yielded high quality RNA, even from a small number of cells (<10k; Figure 5A). To test whether the isolated RNA represented gene expression from a diversity of *P. mytiligera* tissues, we prepared RNA sequencing (RNAseq) libraries from isolated RNA. Following sequencing and mapping to the *P. mytiligera* transcriptome assembly (Gordon et al., 2022; Hendin et al., 2022), we tested whether the gene expression represented cells from multiple tissue types (Methods). We assessed the expression levels of gene markers associated with different cell and tissue types (e.g., muscle, neurons, ciliated cells). We found that tissue specific genes are indeed expressed in our RNAseq libraries (Figure 5B; Supplementary Table S1). However, the small number of cells used for RNA extraction of each library has likely contributed to variability in the observed gene expression. These results indicated that RNA from FACS-purified cells, using this approach, could be used for analyzing gene expression, and therefore facilitate the characterization of different cell populations.

### *In vitro* phagocytosis assay using extracted *P. mytiligera* cells

We next tested whether our cell extraction protocol and flow cytometry approach could be used for functional cell assays. We performed a proof-of-concept *ex vivo* phagocytosis assay. We incubated extracted *P. mytiligera* cells with fluorescent liposomes, containing DiD fluorophore (far red; excitation/emission: 644/665 nm), which could be ingested only by phagocytosis (Methods). Following incubation, cells were analyzed using an imaging flow cytometer (ImageStream; Methods). Background fluorescence level was determined using an unlabeled sample (Figures 5C,D), and fluorescence of free liposomes was determined by using a cell-free sample containing only medium and fluorescent liposomes. The imaging flow cytometer acquired photos of: 1) negative cells: cells negative for far red fluorescence; 2) positive cells: cells having far red fluorescence localized in the cell body; 3) false positives: cells determined as positive by the flow cytometer, according to fluorescence intensity. However, manual inspection of the photo indicated that the liposomes were external to the cell body; 4) free floating liposomes. Further optimization of this assay could reduce the amount of free and cell-attached liposomes. Our results suggested that a fraction of the cells from the anterior body region could uptake large liposomes, and therefore might represent a phagocyte population.

## Discussion

In this study, we developed a method for dissociating *P. mytiligera* tissues and purifying live cells using FACS for further processing. We used this approach for analyzing cells on different flow cytometry instruments, including an analyzer, a sorter, and an imaging flow cytometer. These instruments represent widespread approaches for cell analysis and purification by flow cytometry.

Optimization of tissue dissociation and cell purification required overcoming challenges inherent to this species anatomy, but also relevant to many ascidian species and other marine invertebrates. The thick tunic restricts internal tissue isolation, and body wall tissues are difficult to dissociate without damaging cells. We therefore tested different media conditions for tissue dissociation and examined the disassociation outcome by microscopy. Cellular extraction using enzymatic dissociation of tissue fragments was inconsistent when using high enzyme concentration. This might be a result of variability in tissue composition, as the primary tissues were isolated from different specimens. Alternatively, that might be a result of harsh enzyme activity that affected cell viability. The use of manual mechanical cell extraction resulted in better consistency in cell extraction in our hands, and we therefore used it in subsequent experiments.

Development of a FACS approach for cell purification required autofluorescence analysis, which has been documented in various ascidians (Swalla et al., 1994; Brown et al., 2009; Serrato et al., 2022). We found that commonly used reagents were applicable to *P. mytiligera*, following task-specific optimization. Combination of Hoechst with calcein was particularly effective for isolating live cells, and detection of multiple putative cellular populations. This approach could be applied in future studies to assess the identity and function of uncharacterized cell populations. Further optimization of Hoechst labeling could be utilized in the future to separate cells based on their DNA content (Kim and Sederstrom, 2015), as a proxy for isolating cells based on their cell cycle state. This would be extremely useful for understanding the cellular basis of regeneration (Gordon et al., 2021). In planarians, highly regenerative flatworms, pluripotent stem cells mediate regeneration (Wiggans and Pearson, 2021; Pearson, 2022). In *P. mytiligera*, regeneration involves cell proliferation (Gordon et al., 2021), but the cells that promote this process have not been identified yet. Therefore, the methods developed here facilitate studying the mechanisms of regeneration in the *P. mytiligera* system.

We showed here two proof-of-concept experiments that require tissue dissociation and cell purification. First, we applied RNAseq to FACS-purified cells. Genes likely expressed in different cell types were represented in the dataset. However, the libraries were prepared from RNA extracted from a small number of cells (∼10k), and therefore were likely not able to capture the true complexity of the analyzed tissues. Preparation of RNAseq libraries using larger amounts of input RNA, or the use of alternative approaches (e.g., single cell RNAseq), should be considered for comparative gene expression analyses of *P. mytiligera* purified cells. In a second proof-of-concept experiment, we analyzed the cellular uptake, *ex vivo*, of fluorescent particles. Comparison of cells incubated with the reagent with control cells showed a major increase in signal intensity in the treated cells. This suggests that a similar assay could be used for studying phagocytosis in *P. mytiligera*.

## Conclusion

Tunicates are a powerful system for exploring the evolution of chordate regeneration. Yet, live cell isolation requires system-specific optimization, which could require significant resources. The ability to isolate diverse live cell populations from *P. mytiligera* is a significant step towards establishment of cell function assays. We have shown here that isolated *P. mytiligera* cells could be used for downstream applications. We anticipate that application of single cell transcriptomics to isolated *P. mytiligera* cells could be extremely valuable for an initial characterization of *P. mytiligera* cell types, for profiling their responses to injury, and comparing these with other organisms.

## Methods

### Animal collection and maintenance

Animals were collected by SCUBA diving in the bay of Aqaba (Eilat) and transferred to Tel Aviv University. Animals were maintained in a recirculating aquarium system with artificial sea water (Red Sea Salt, 8 kh) mixed in reverse osmosis water.

### Media used for testing tissue dissociation

The following media compositions and enzymes were tested for *P. mytiligera* tissues dissociation: 1) NaCl (449 mM), Na_2_SO_4_ (33 mM), KCl (9 mM), NaHCO_3_ (2.15 mM), EDTA (292.24 mM), Tris-Cl (5 mL), pH range tested between 8 and 8.2, concentration part per trillion (ppt) 32. This medium was used in combination with trypsin (0.25%, Sartorius, 03-052-1A) and collagenase (1 mg/mL, Sigma, C0130) added 1:10. 2) NaCl (0.4 M), KCl (10 mM), MgSO_4_-7H_2_O (7.6 mM), MgCl_2_-6H_2_O (52 mM), Na_2_SO_4_ (21 mM), NaHCO_3_ (3 mM), SrCl_2_ (0.17 mM), EDTA (5 mM). pH 8, ppt 40. This medium was tested by adding collagenase (1 mg/mL) to 1:10 and 1:20. 3) PBS x3.3, pH 7.5, ppt 35. This medium was tested by adding collagenase (1 mg/mL) 1:20 and trypsin (0.25%). The efficiency of the different combination of medium and enzymatic activity was determined by analyzing the percentage of viability dye (propidium iodide, PI) (1:1000) positive cells using hemocytometer.

### Optimized tissue dissociation procedure

Tissue fragments of up to 3 cm were isolated surgically from adult animals using a blade. Following tunic removal, the tissue fragments were washed with filtered (0.2 μm) artificial sea water (ASW; 0.4 M NaCl, 10 mM KCl,7.6 mM MgSO_4_-7H_2_O, 52 mM MgCl_2_-6H_2_O, 21 mM Na_2_SO_4_, 3 mM NaHCO_3_, 0.17 mM SrCl_2_, 10 mM HEPES, 5 mM EDTA, pH 8, 40 ppt) and transferred into a sterile Petri dish on ice. Using a blade, the tissue fragments were cut to smaller fragments, centrifuged at 300G at 4 °C for 5 min and resuspended in 1 ִml artificial sea water. For the enzymatic treatment samples were incubated with enzymes (collagenase I, Sigma, C0130) for 7 min at room temperature (RT) and mixed by pipetting. Then 1 mL of cold ASW with 0.5% BSA (Mercury, 821006) was added to the sample. The sample was then filtered through a 40 μm mesh filter (LifeGene, G-CSS010040S). Cell suspensions were further dissociated on the mesh by using a sterile plunger of 1 mL syringe (PIC, 00603308), washed, and collected in ASW. Then, cells were collected by centrifugation at 300G at 4°C for 7 min, and then resuspended in 1 mL ASW with 0.5% BSA. Cells were labeled using the nuclear dyes, Hoechst 33342 (Thermo Fisher, H3570) and DRAQ5 (Abcam, ab108410), to a final concentration of 20 μM.

Calcein-AM (BioLegend, #425201) and Calcein Violet-AM (BioLegend, # 425203) were used as live cell labeling dye by supplementing to a final concentration of 4 μM. Cells were incubated for 30 min at room temperature. Cell concentration was estimated using a hemocytometer on an inverted confocal microscope system (Zeiss LSM800).

### Cell sorting, flow cytometry analysis, and live cell microscopy

FACS reading was done on BD FACS Aria II, and gating for cell sorting was done using the BD FACSDiva™ software (Becton Dickinson Biosciences). Flow cytometry analysis was done using Beckman Coulter CytoFLEX 4L (Beckman Coulter). Imaging on flow cytometry was performed using the ImageStream^x^ mk II platform (Cytek). For determining the gating of cells and debris, sorting and observation by confocal microscopy was done several times. Analysis of flow cytometry data was done using Kaluza Analysis Software (Beckman Coulter) and IDEAS^®^ 6.2 ImageStream Analysis Software. Specification of excitation laser and optical detection filter for emission for each analysis is shown in the figures. The excitation laser was measured in nm and filters are stated as long pass (LP) and band pass (BP).

Cell sorting was performed with 100 μm nozzle size and sorted directly into Eppendorf tubes containing 100 μL of ASW in order to minimize cellular stress. Cells (10,000–50,000) of each population of interest were sorted at a speed of 1500 cells/second.

For live cell microscopy, sorted cells were collected by centrifugation at 4°C; 300G; 10 min. The cells were resuspended in 20 μL of ASW and counted using a hemocytometer. Images of sorted cells were acquired using confocal microscopy (Zeiss LSM800) using the Zeiss Zen Blue v2.3 software. In addition, fluorescence microscopy was used to image autofluorescence in unlabeled cells (Leica SP8, Laser: 488 Filter: 500–560; Laser: 561 Filter: 571–639; Laser: 633 Filter: 645-730).

### Phagocytosis assay

Cells were extracted from the anterior body region of animals following tunic removal. Extracted cells were resuspended in 800 µL of L-15 medium containing glucose and Fetal Bovine Serum (Sigma Aldrich, F4135) in a 24-well plate. For the treatment groups, the medium was supplemented with 8 μL of liposomes containing the fluorophore DiD (Encapsula Nanosciences, CLD-8904), with excitation and emission spectra 644 and 665 nm, respectively. Incubation in medium containing fluorescent liposomes was performed for 4.5 h at room temperature in the dark. Prior to analysis using an imaging flow cytometer (ImageStream MKII), the medium was supplemented with 4′,6-diamidino-2-phenylindole (DAPI) for 30 min (1 mg/μL).

### RNA quality assessment and extraction

RNA extractions were performed using TRIzol LS (Thermo Fisher; #10296010), following the manufacturer’s protocol. RNA concentration was measured by RNA fluorometry (Qubit 4, Life Technologies) using the RNA high sensitivity kit (Q32852). RNA integrity was assessed using a microcapillary electrophoresis (TapeStation 4200, G2991BA) with RNA ScreenTape kit (Agilent Technologies, #5067-5576).

### RNA sequencing library preparation

Illumina-sequencing compatible RNAseq libraries were prepared using New England Biosciences (NEB) NEBNext Ultra II Directional RNA mRNA seq library (NEB; #E7760L) according to the manufacturer protocol, with at least 10 ng total RNA per library, and an estimated number of cells in the range of 10,000. Briefly, mRNA was enriched by polyA selection using poly-dT paramagnetic beads included in the kit. Then, RNA was fragmented according to the protocol, and complementary DNA (cDNA) was produced by reverse transcription. Following second-strand synthesis, the resultant DNA was end-repaired. Adapter ligation was performed according to protocol. The libraries were then amplified by PCR using barcoded primers according to the kit instructions. Quantitative PCR (qPCR) with Illumina primers was used to determine the optimal number of PCR amplification cycles. The number of amplification cycles (17-20) represented an early exponential increase in the qPCR signal. Following PCR amplification, libraries were sequenced using Novogene sequencing services.

### RNAseq libraries analysis

RNAseq libraries were processed similarly to previously described (Hendin et al., 2022). Briefly, RNAseq libraries were trimmed using cutadapt v2.85 using the Illumina adapter sequences (Martin, 2011). Then, the preprocessed library reads #1 (first read in pair) were mapped to a transcriptome assembly of *P. mytiligera* using bowtie2 v2.4 using flags -local--trim3 80 --trim5 2 --sensitive-local (Langmead and Salzberg, 2012). A gene expression matrix was produced using featureCounts package v2.0.0 with parameters allowing multi mapping [-M -s 0] (Liao et al., 2014). Gene expression levels in the libraries were estimated using DESeq2 and expression levels, across libraries, were normalized using the variable stabilizing transformation in the DESeq2 package. Genes expressed in a cell type-enriched or tissue-enriched manner were extracted from the GeneCards database manually using tissue and cell type keywords (Stelzer et al., 2016), and similar sequences in *P. mytiligera* were determined by using BLAST search for the best blast hit with e-value <10^–20^ (Camacho et al., 2009).

## Data Availability

RNAseq data was deposited in the Sequence Read Archive (SRA) (Leinonen et al., 2011) under accession PRJNA996490, https://www.ncbi.nlm.nih.gov/sra/PRJNA996490.

## Supplementary material

Supplementary Figure S1 Cell isolation using different extraction media.

Supplementary Table S1 Gene expression in RNA-sequencing libraries of contigs having BLAST hits against UNIPROT.
