# Supplementary material for: Methods for cell isolation and analysis of the highly regenerative tunicate Polycarpa mytiligera
Source: Front Cell Dev Biol. 2023 Oct 11;11:1274826. doi: 10.3389/fcell.2023.1274826 (PMC10598751; doi:10.3389/fcell.2023.1274826)
Supplement: Supplementary file 1 [file DataSheet1.PDF]

Supplementary Material

Methods for cell isolation and analysis of the highly regenerative tunicate *Polycarpa mytiligera*

Tal gordon, Noam Hendin, Omri Wurtzel*

*** Correspondence: owurtzel@tauex.tau.ac.il**

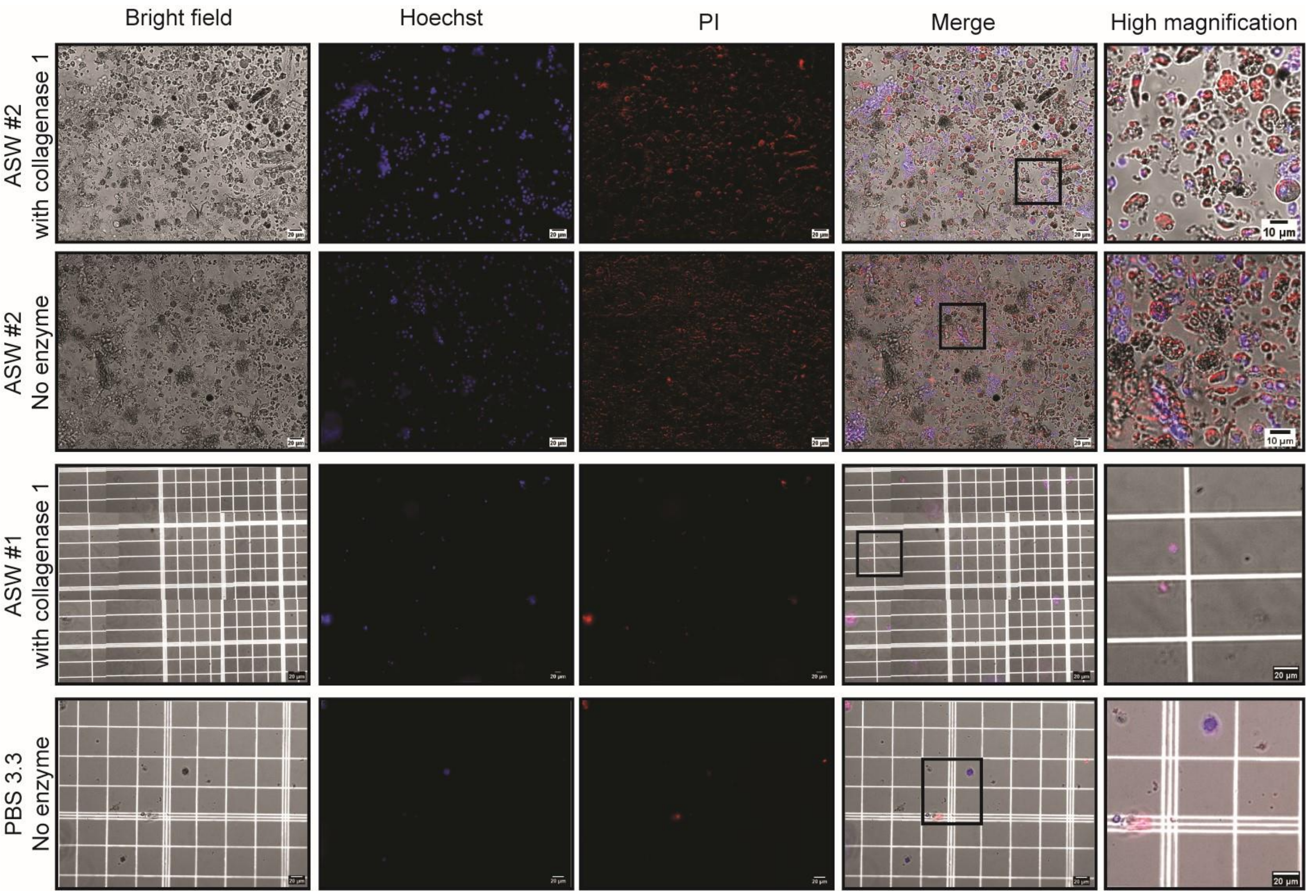

Supplementary Figure 1. *P. mytiligera's* isolated cells following tissues dissociation using different media compositions and enzymatic treatments. Cells were labeled with nuclear dye (Hoechst) and viability dye (propidium iodide, PI). The use of ASW #2 medium was favorable, either when used with collagenase 1 or without enzymatic treatment. Using PBS or ASW #1 as the dissociation media resulted in extremely poor cellular recovery and cell viability (Methods).
